# Exploration for Thermostable β-Amylase of a *Bacillus* sp. Isolated from Compost Soil to Degrade Bacterial Biofilm

**DOI:** 10.1128/Spectrum.00647-21

**Published:** 2021-10-06

**Authors:** Ieshita Pan

**Affiliations:** a Department of Biotechnology, Saveetha School of Engineering, Saveetha Institute of Medical and Technical Sciences, Saveetha University, Chennai, Tamil Nadu, India; University of Guelph

**Keywords:** amylase, fermentation, optimization, characterization, biofilm

## Abstract

In an attempt to explore biofilm degradation using extracellular amylase, a potent amylase-producing bacterium of compost origin, B. subtilis B1U/1, was found to grow suitably in a simple medium of pH 7.5 for 48 h at 37°C under agitation of 140 rpm. This bacillary amylase was recovered by ammonium sulfate precipitation and purified to near homogeneity by membrane filtration and DEAE cellulose column chromatography. The amylase was purified to 4.5-fold with almost 50% yield and 26 kDa of molecular weight. Stable enzyme activity was found in a pH range of 5.2 to 9.0, while 90% residual activity was recorded at 90°C, indicating its thermostable nature. In the presence of 1 mM Fe^++^ and Ca^++^, the activity of amylase improved; however, it is inhibited by 1 mM Cu^++^. In the presence of 5% NaCl concentration, amylase showed 50% residual activity. The end product analysis identified the enzyme as β-amylase, and a crystal violet assay ensured that it can degrade Pseudomonas aeruginosa (78%) and Staphylococcus aureus biofilm efficiently (75%). The experiments carried out with the compost soil isolate were promising not only for biotechnological exploitation due to its pH flexibility during growth but also for high efficiency in the degradation of biofilms, which makes the organism a potent candidate in the fields of food industries and biomedical engineering, where it can be used as a prosthetic and hip joint cleaner. The β-amylase is highly thermostable since it withstands an elevated temperature for a prolonged period with a minimum loss of activity and is also moderately salt and metal tolerant.

**IMPORTANCE** More than 85% of nosocomial infections are due to the development of bacterial biofilms. Recent research proposed that biofilm-like structures are not only visible in autopsies, biopsies, patients with chronic wounds, and exudates in animal models but are also present in biomedical devices, implants, prosthetic valves, urinary catheters, etc. Because complete eradication of biofilm is highly challenging, alternative methods, such as enzymatic damage of extracellular matrix and mechanical removal, are being implemented due to their easy availability, low cost, and high yield. Organisms from compost piles are rich sources of diverse extracellular enzymes with a high level of stability, which makes them able to withstand the different conditions of their environments. Under diverse environmental conditions, the enzymes are active to continue degradation processes, making them potential candidates in waste management, medicine, and the food and agriculture industries.

## INTRODUCTION

Recently, food industries have focused on the best technology to eliminate bacterial biofilms, which are the complex microbial networks produced by either a single or multiple bacterial inoculations enclosed with an extracellular matrix (ECM), where the composition depends on the food manufacturing environment and the type of colonizing bacteria (1). Among those organisms, Bacillus cereus (exudes toxins for diarrhea and nausea), Salmonella enterica (child death), Escherichia coli (enterotoxigenic E. coli [ETEC] and enterohemorrhagic E. coli [EHEC]), Staphylococcus aureus (enteric toxins), and Listeria monocytogenes (responsible for abortion during pregnancy) are predominating (2). In order to eliminate the organism that makes these harmful biofilms, the common, widely used, and acceptable strategies include nano-composite application, inhibition of cell-signaling, chemical healing, nonthermal plasma therapies, bacteriophage P100 application, use of nisin (bacteriocins) and biosurfactants (surfactin), and extraction and application of essential oils such as citral, tea tree extract, or carvacrol. In medical devices, too, nosocomial infections begin when bacteria colonize the material surface of the biomedical device, which quickly complicates the transformation of the resident microbial community into a biofilm producer (3). The present study focused on enzymatic disruption of ECM.

Soil is the major component of terrestrial ecosystems and is the most fundamental constituent of natural resources, as it supports all terrestrial life forms. Higher-order activities influence growth and colonization in soil, which further influences the soil microenvironment. Thus, soil may harbor potential microorganisms capable of hydrolyzing polymers. Such hydrolytic capability of microorganisms would influence organic matter decomposition (4). Composting, an organic matter decomposition system, is a three-phase aerobic or anaerobic biological process, dominated by hydrolyzing microorganisms to work even at 75°C (5). Among the different hydrolytic activities of the microorganism, amylolysis is consistently common (6–9). Though there may be several sources, bacterial amylases are found to be more thermostable (7).

Amylase is one of the largest families of enzymes, with about 30 enzyme specificities. Based on hydrolytic abilities (10), they are of two types, α- and β-amylases. The α-amylases (EC 3.2.1.1), which degrade starch to soluble maltodextrins, maltose, and glucose, are endoenzymes, whereas β-amylase (EC 3.2.1.2) cleaves the nonreducing chain termini, which produces incomplete hydrolysis, yielding maltose, and limits dextrins and therefore acts as the exo-acting enzymes (11, 12).

Although there is a lot of information regarding fermentation and amylolytic activity, the exploration of compost soil seemed to have scanty information. The literature reveals a vast microbial amylase resource that encompasses the necessity for further exploration (10, 13). The current study was undertaken to explore the removal of pathogenic bacterial biofilm in medical and food industries using thermostable amylase from B. subtilis B1U/1 (isolated from compost soil), which can actively withstand the entire process of composting, enzyme production, optimization, purification, and characterization.

## RESULTS

### Optimization of amylase production.

**FIG 1:**
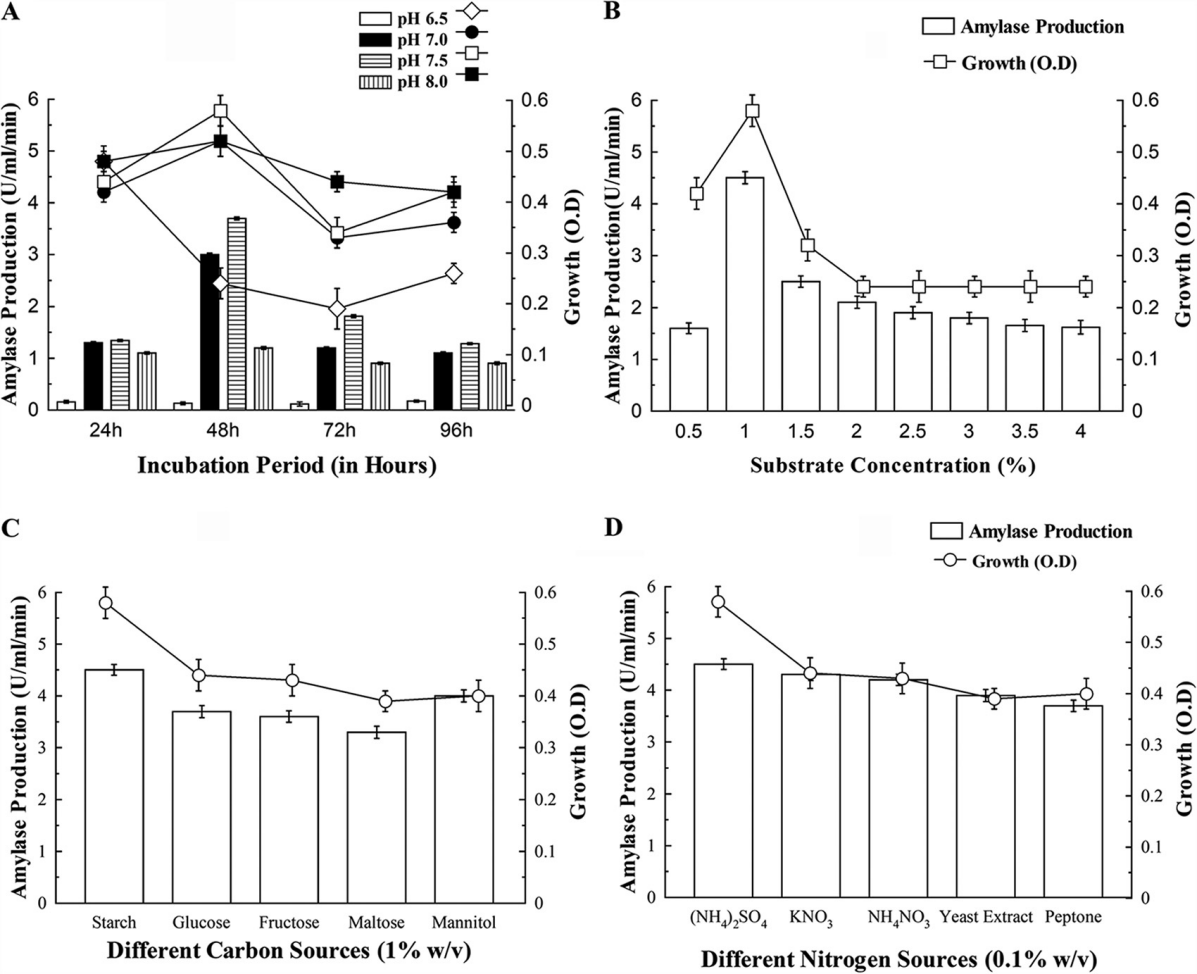
Growth and extracellular amylase production by B. subtilis B1U/1. (A) Effect of different pHs and time periods. (B) Effect of substrate concentration at pH 7.5 after 48 h of incubation. (C) Effect of carbon sources. (D) Effect of nitrogen sources. Bars represent enzyme production (U/ml/min), and the line graph represents bacterial growth. Results are the mean of three independent experiments ± the SD.

Experiments were conducted to explore the amylase production by Bacillus subtilis B1U/1, isolated from soil compost. To determine the suitable incubation period for amylase production with isolate B1U/1, growth and production parameters were carried out after 24-h intervals (Fig. 1A). The results showed that the isolate grew well and produced extracellular amylase throughout the experimental period but in varied amounts. The growth and enzyme production increased up to 48 h and decreased thereafter with the increase of time. However, for the maximum production of enzyme, the working isolate, B. subtilis B1U/1, was agitated at 140 rpm during the entire study.

Extracellular amylase production was also determined at a range of pH (pH 6.5 to 8.0), where significant growth and enzyme production were obtained. The optimum pH for amylase production (3.7 U/ml/min) was found to be pH 7.5 and 48 h of incubation (Fig. 1A).

The effect of starch on growth and amylase production showed no positive effect of growth; however, there was a significant increase in amylase production (4.5 U/ml/min) at 1% starch concentration (Fig. 1B). The growth, as well as enzyme production (1.62 U/ml/min), decreased with the increase of starch concentration, indicating substrate inhibition. Also, a 22% decrease in amylase production was observed when the starch substrate was replaced with maltose as the carbon source (Fig. 1C).

To understand the suitable source of nitrogen for the bacterial growth and enzyme production, both common organic and inorganic sources were tested at 0.1% in the minimal medium. Studies showed a mixed trend in amylase production, which was maximized in the presence of ammonium sulfate (4.5 U/ml/min), whereas with peptone, both growth and enzyme production were inhibited significantly, suggesting the inability of the bacteria to utilize complex nitrogenous compounds (Fig. 1D).

### Characterization of amylase.

**FIG 2:**
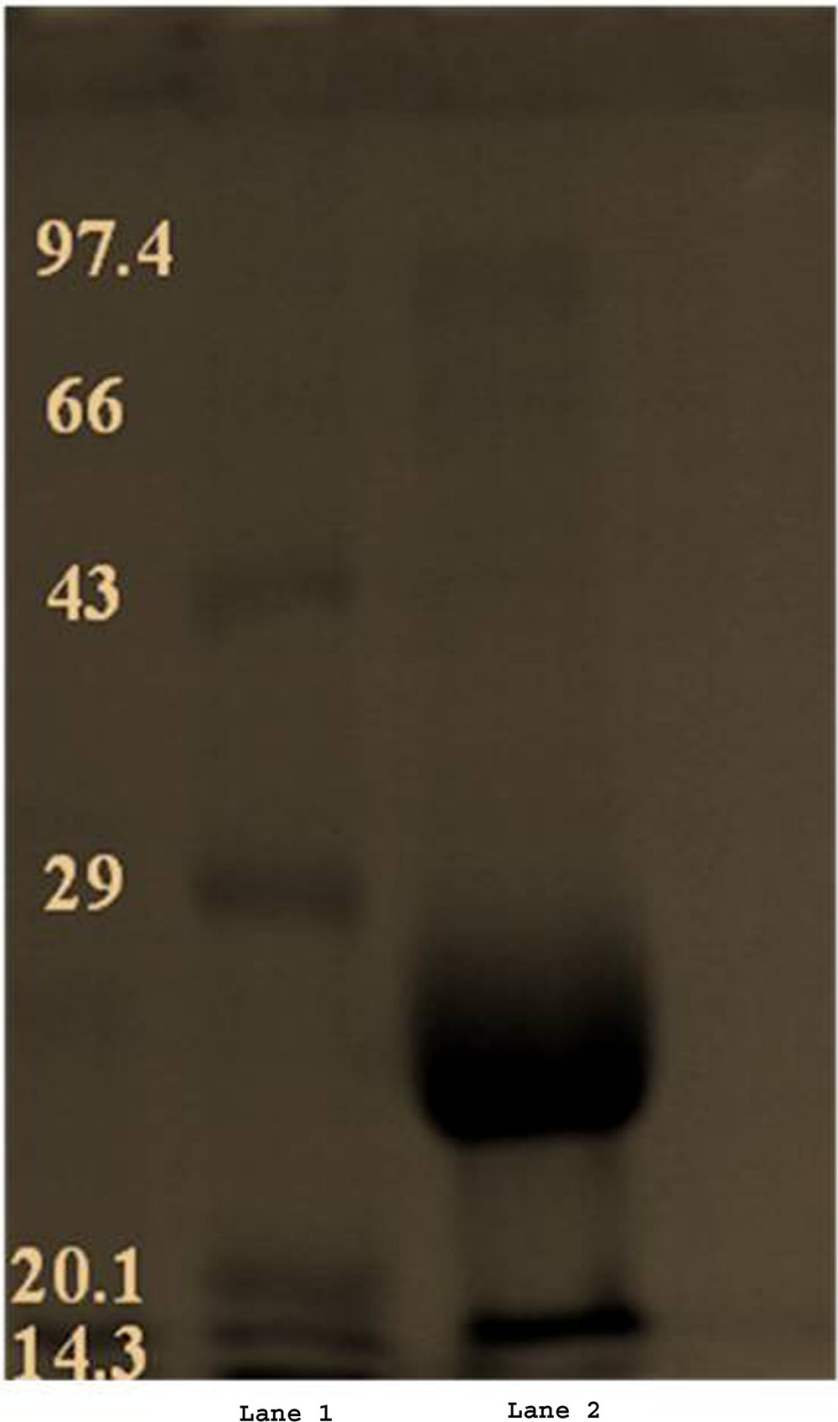
SDS-PAGE analysis of β-amylase. (Lane 1) Molecular weight marker (GeNei; catalog no. 623110275001730); (lane 2) sample.

The enzyme was recovered with ammonium sulfate (80%) precipitation and centrifuged, and the pellet was dissolved in phosphate buffer of pH 7.6. The solution was further purified through dialysis using the same buffer. The dialyzed and partially purified enzyme solution, loaded on an ion-exchange chromatography instrument, gave a 4.48-fold purified bacillary amylase with a specific activity of 31.62 U/mg with 49.18% yield and total protein of 7.0 mg (Table 1). SDS-PAGE analyses of the amylase showed a single band with a molecular weight estimated to be 26 kDa (Fig. 2).

**TABLE 1:** Purification of β-amylase from Bacillus subtilis B1U/1

Purification step	Enzyme solution (ml)	Total protein (mg)	Total activity (units)	Sp. act (units/ mg of protein)	Purification fold	Yield (%)
Crude extract	100	63.80	450.00	7.05		100
Enzyme precipitate	70	31.51	350.00	11.10	1.57	77.77
Dialysis	30	17.10	255.00	14.90	2.11	56.66
DEAE cellulose	15	7.00	221.34	31.62	4.48	49.18

**FIG 3:**
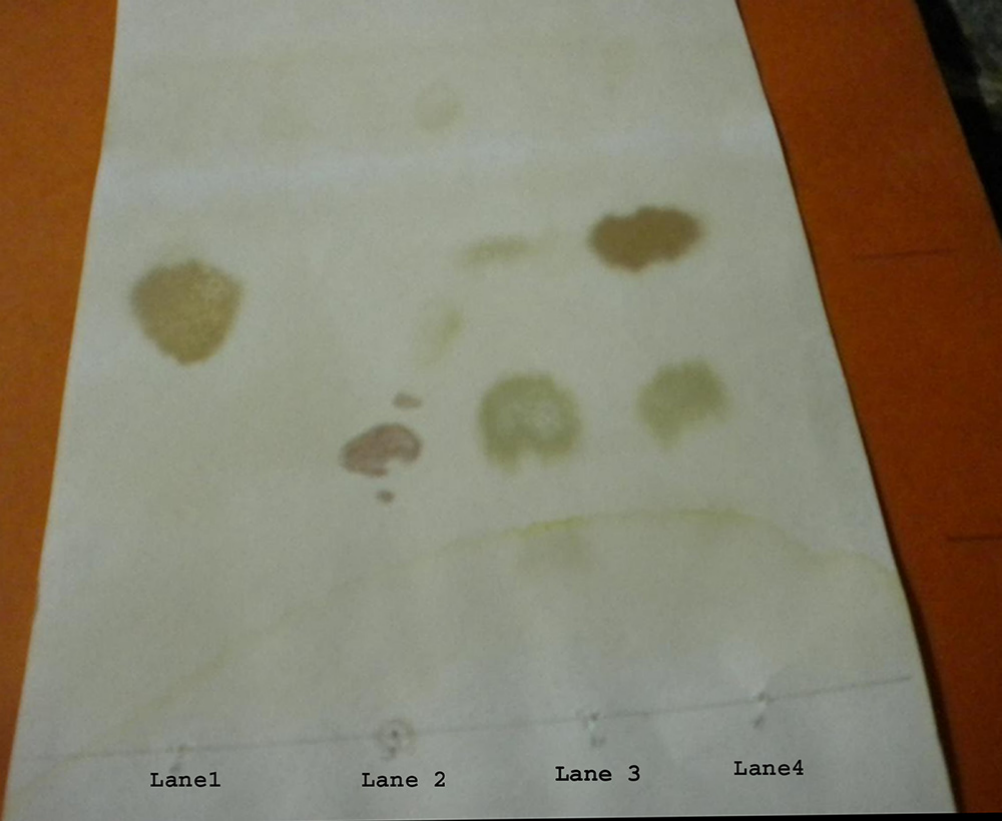
End product analysis of enzyme substrate reaction by thin-layer chromatography. (Lane 1) Standard glucose; (lane 2) standard starch; (lane 3) standard maltose; and (lane 4) enzyme substrate mixture.

For resolving the nature of amylase, the enzyme-substrate mixture reacted with Lugol’s iodine solution gave a violet color which later changed to consistent purple, whereas standard α-amylase reacted with the substrate produced an initial violet color which became colorless after few minutes. Also, the end products of the enzyme-substrate reaction showed the presence of glucose and maltose upon chromatographic analysis (Fig. 3). Thus, the studied enzyme is assumed to be β-amylase.

**FIG 4:**
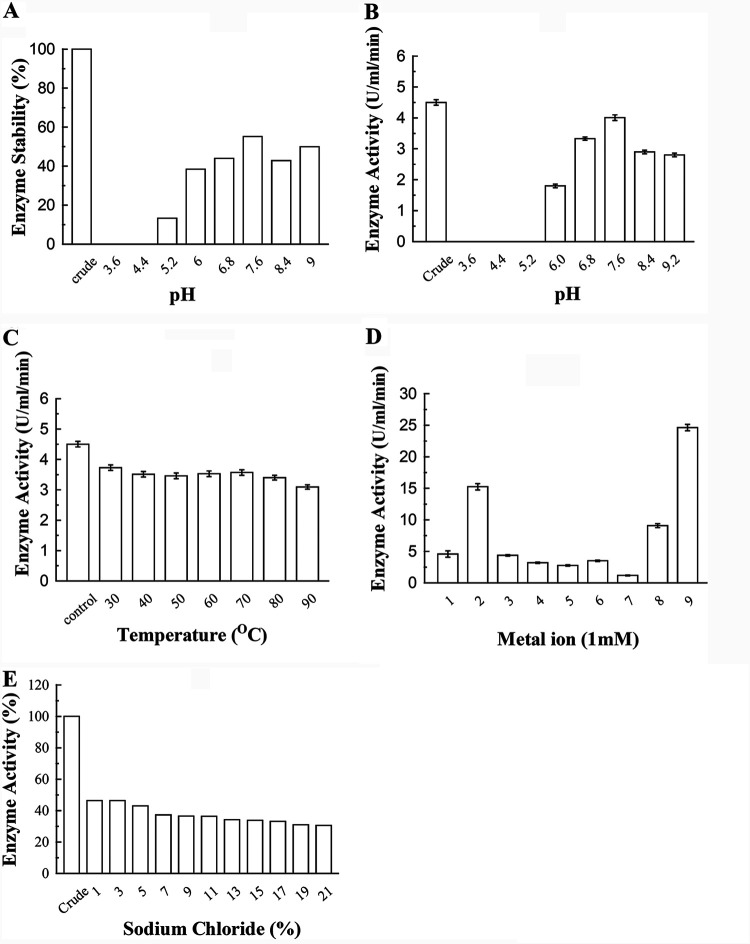
Characterization of the purified enzyme from B. subtilis B1U/1. (A) Effect of pH on amylase stability. (B) Effect of pH on amylase activity. (C) Effect of temperature on amylase stability. (D) Effect of heavy metal on amylase activity. Bar 1–9 represents: 1: Crude enzyme; 2: CaCl_2_; 3: NiCl_2_; 4: MnSO_4_; 5: ZnSO_4_; 6: MgSO_4_; 7: CuSO_4_; 8: NaCl; 9: FeSO_4_. (E) Effect of sodium chloride on amylase activity. Bars represent the enzyme activity. Results are the mean of three independent experiments ± the SD.

The effect of pH on amylase stability was determined by incubating the enzyme (1 ml) with a specific buffer (1 ml) containing substrate (starch, 1%) at 4°C for 24 h. The results showed that at least 50% activity was retained at a pH range of 5.2 to 9.2 (Fig. 4A). Enzyme activity increased (40.0% to 89.1%) between pH 6.0 and 7.6 but gradually decreased to 62% and maintained a steady state, showing the optima at pH 7.6. At this pH, approximately 56% enzyme recovery was achieved. The amylase activity was inhibited at pH below 5.2, as only 14% enzyme activity was obtained. This indicated that the β-amylase was stable at a broad pH range as shown in Fig. 4B.

To understand the effect of temperature on stability, the enzyme solution was treated at a different temperature range, 30 to 90°C for 1 h and was reacted with the substrate under standard conditions. The enzyme was found to be stable at a different tested temperature range and showed 90% stability at a temperature of 90°C for 1 h (Fig. 4C). In comparison, the amylase of working isolate B1U/1 was found to be stable even at a lower temperature (<90°C), but the detected residual activity higher with prolonged temperature treatment, suggesting a higher degree of thermostability of β-amylase.

The enzyme activity was also evaluated in the presence of selected metal ions at a final concentration of 1 mM. The results showed that the enzyme’s activity increased significantly in the presence of Ca^++^ and Fe^++^ but decreased significantly in the presence of Cu^++^ (Fig. 4D). Also, using 5% sodium chloride, 50% amylase activity was obtained, and upon further increases in NaCl concentration, the enzyme activity was inhibited gradually (Fig. 4E). The studied amylase withstood up to 15% NaCl, showing 30% activity, which suggested a moderate halotolerant property.

### Application of β-amylase to degrade bacterial biofilms.

**FIG 5:**
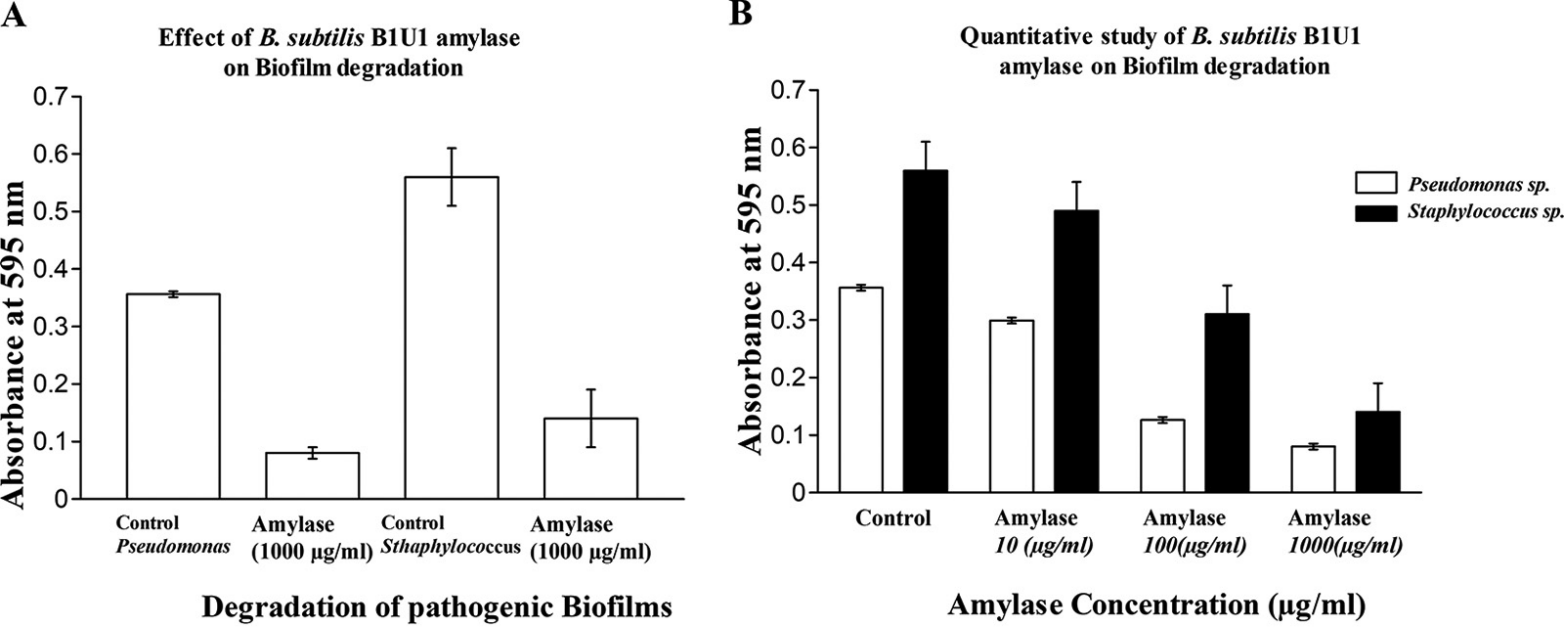
Biofilm degradation by amylase isolated from B. subtilis B1U/1. (A) Effect of Bacillus subtilis B1U1 amylase on biofilm degradation. (B) Quantitative study of B. subtilis B1U/1 amylase on biofilm degradation.

To understand the effect of amylase on biofilm degradation, S. aureus and P. aeruginosa biofilms were grown and observed with a modified crystal violet assay (Fig. 5A). The development of Pseudomonas and staphylococcal biofilm was studied in LB broth for 72 h, and the degradations were analyzed upon amylase application. B. subtilis B1U1 amylase degraded both Pseudomonas and Staphylococcus biofilms. For Pseudomonas, degradation of biofilm was recorded up to 16%, 65%, and 78% on treatment with 10, 100, and 1,000 μg/ml enzyme application; however, for Staphylococcus, biofilm degradation was estimated up to 13%, 45%, and 75% on those applied enzyme concentrations (Fig. 5B).

## DISCUSSION

### Enzyme production.

Earlier studies revealed that amylase production was maximum at 52 h, during the rapid-growth phase (10). However, B. subtilis B1U/1 reached its maxima within 48 h of incubation but well within the exponential-growth phase. A closely comparable trend of amylase production was observed with a *Bacillus* sp. isolated from cassava waste (8, 14). Production of bacterial amylase was maximized between pH 4.8 and 9.2 by Bacillus licheniformis isolated from cassava waste (8). Also, Valaparla (12) observed maximum amylase production at a pH range between 3.0 and 9.0. The growth of *Bacillus* spp. was optimum under agitation at 200 rpm (14), but enzyme production was maximized at 240 rpm (12). In addition, optimal amylase production was recorded at reduced agitation (150 rpm) (10). The present study demonstrated both growth and enzyme production under much lower (140 rpm) agitation.

Upton and Fogarty (10) observed maximum amylase production with 1.5% (wt/vol), a much higher concentration of starch. The result in which bacterial growth and enzyme production decreased with an increased concentration of starch is typically corroborated by the work of Nipkow et al. (15) and Aygan et al. (14). Asgher et al. (16) emphasized that not only starch, but also the accumulation of organic acids and other metabolites, changed the environmental conditions which facilitated growth rate inhibition and enzyme production. Since ammonium sulfate served as a suitable nitrogen source, the influence of sulfur, indicating rapid growth and enzyme production, thereby reciprocated as an extended exponential phase and resulted in elevated biomass and improved enzyme yield.

### Enzyme purification and characterization.

Obi and Odibo (17) observed 31.6 kDa of β-amylase isolated from actinomycetes. The differences in molecular weights of β-amylases indicated genomic variation among the organisms (14, 18, 19). As a protease inhibitor, phenyl methane sulfonyl fluoride (PMSF) did not exert a negative effect on amylase activity or on the enzyme stability under standard assay conditions. Moreover, detection of maltose as a major component from the hydrolyzed product confirmed its nature as β type (17). It has been reported that most bacilli producing amylase have optimum temperature stability between 40 and 70°C (14, 20–22). Obi and Odibo (17) reported that β-amylase withstood up to 70°C, while Fogarty and Griffin (23) observed temperature tolerance of amylase up to 52°C (Bacillus polymyxa) and 70°C from another *Bacillus* sp. (16). However, Poddar et al. (24) reported full activity of β-amylase from B. subtilis DJ5 at 100°C for 15 min of treatment. Takasaki (25) reported amylase stability at near-neutral pH, but the bacillary amylase from B1U/1 was found to be stable at a very wide range of pH.

For enzyme activity and structural integrity of amylase (the metalloenzyme), Ca^++^ was much stronger than any other ions (16, 26, 27). Under the experimental conditions, the isolate B1U/1 showed enhanced enzyme activity in the presence of Ca^++^, higher than to Cu^++^ and much lower than Fe^++^, signifying affinity to a specific metal ion. In contrast, Cordeiro et al. (21) observed inhibition of the amylase activity in the presence of Ca^++^, and competition between exogenous cations with protein-associated cations might be the reason for this inactivation. Similarly, Takasaki (25) did not record any stimulation of activity by any of the metal ions to β-amylase of Bacillus cereus var. *mycoides*. Asgher et al. (16) and Bano et al. (27) recorded inhibition of amylase activity in the presence of Fe^++^. However, in the presence of Cu^++^, the activity of amylase enzyme was found to decrease, which suggested selective toxicity of β-amylase activity (28). Aygan et al. (14) reported NaCl tolerance up to 10% of bacillary amylase.

### Biomedical application of B1U/1 amylase.

Bacteria present in biofilms have several characteristics which made them difficult to completely eradicate. Furthermore, the biofilm matrix is often predominated by several factors derived from hosts, proteins secreted and lysed, polysaccharides, and DNA. Del Pozo and Patel (29) explained that due to the inherent antibiotic tolerance of resident bacteria, it is difficult to eradicate biofilm diseases, which made an antimicrobial treatment a failure. Thus, the only option is to remove the biofilm either from a food source or from biomedical devices (e.g., implanted device, wound debridement, etc.). Hydrolysis of extracellular matrix components present in biofilms by several enzymes, which makes them nonviable, is thus the best way to prevent disease establishment. Once the biofilm matrix is partially damaged, either mechanical treatments or the application of sanitizers can remove them completely (30). Trizna et al. (31) reported that 50 to 60% degradation was recorded with enzymes such as papain and trypsin (1 mg/ml).Compared with the existing knowledge, the present study showed similar findings with regard to α-amylase, reported by Craigen et al. (32), and DNase I, reported by Tetz et al. (33). The β-amylase from B. subtilis *B1U1* was found to be efficient not only due to its degradation capability within a very short time but also due to its working potential at 100 times lower concentration.

### Conclusion.

The B. subtilis B1U/1 isolate from compost soil was found to produce significant amounts of β-amylase (4.5 U/ml/min) under the following conditions: 1% starch substrate, 48 h of incubation at 37°C, pH 7.5, inoculum size of 10^9^ CFU/ml, and agitation at 140 rpm. The β-amylase enzyme showed a stable activity under a broad pH range, and activity of 90% was recorded at 90°C for 1 h. Furthermore, the enzyme was halotolerant and hyperactive (>3-fold increment) in the presence of Ca^++^ and Fe^++^. Purification of the enzyme gave a 4.5-fold and 50% yield. A test of the ability of the B. subtilis B1U/1 amylase to degrade bacterial biofilms showed that the enzyme effectively cleaned up the Staphylococcus and Pseudomonas biofilms at 1,000 μg/ml concentration.

## MATERIALS AND METHODS

### Optimization of fermentation conditions.

As a potent amylase producer, compost soil isolate B1U/1 was characterized up to the molecular level and identified as B. subtilis B1U/1 (GenBank accession no. GU723510 (34). The isolation was done by enrichment of soil with carboxy methyl cellulose (34), followed by serial dilution pour plating (35). The growth medium (g/liter) comprised starch (1.0), NaNO_3_ (2.0), K_2_HPO_4_ (1.0), MgSO_4_, 7H_2_O (0.5), KCl (0.5), peptone (2.0), and agar (15.0) at pH 7.0; this was incubated at 37°C for 48 h. The isolate was maintained in the same medium at 4°C. The basal medium (36) (g/liter) comprised KH_2_PO_4_ (3.0), sodium citrate (0.5), K_2_HPO_4_ (7.0), MgSO_4_, 7H_2_O (0.1), (NH_4_)_2_SO_4_ (1.0), and starch (1.0). To determine the optimum fermentation period (37), the basal medium was inoculated with 24-h-old broth (2%, 10^9^ CFU/ml) and fermented up to 96 h at 37°C, under shaking conditions (140 rpm). The effect of pH on fermentation was checked using different buffer systems of varied pH values, between pH 6.5 and 8.0, with a gradual increase of 0.5. To optimize the suitability of the carbon concentration, experiments were executed using different quantities of starch (0.5 to 4%). Also, further supplementations using carbon and nitrogen sources were carried out.

### Enzyme assay.

The assay mixture contained 1 ml of extracellular enzyme solution with 1 ml of soluble starch (1% wt/vol). After adding 1 ml of 3,5-dintro salicylic acid (DNS) reagent, the reaction mixture was kept in a water bath at 90°C for 5 min, and the amount of reducing sugar released was determined at 540 nm (38).

A unit of enzyme is defined as the quantity of 1 mM glucose released at 90°C for 1 min from the substrate and is expressed as U/ml/min. Quantification was done using glucose as the standard.

### Biofilm degradation.

To check the outcome of bacillary amylase on the degradation of pathogenic biofilm, LB medium (pH 7) was inoculated with 2 ml of fresh S. aureus (Himedia; TKC030) and Pseudomonas aeruginosa (Himedia; TKC031) culture with 0.1 OD (optical density at 600 nm [OD_600_]) separately in sterile glass tubes, and the mixture was incubated for 72 h at 37°C for the formation of biofilm. After 72 h, the existing medium removed from each tube and replaced with fresh amylase (5 μg/ml) containing LB broth, and this was further incubated for 24 h. A tube with LB medium without enzyme was used as the control for this study. A Congo red solution (50 μg/ml final concentration) was used for biofilm staining (39).

Biofilm quantification was performed with a modified crystal violet assay (40). In this modified crystal violet assay, pathogens were grown in sterile tubes under static conditions for 72 h to achieve good growth as in the Congo red method. Upon growth of the pathogen, the existing medium was substituted with fresh LB containing amylase with concentrations of 10 μg/ml, 100 μg/ml, and 1,000 μg/ml and was further incubated for 24 h at 37°C. Then the culture supernatant was gently decanted, and the tubes were washed with phosphate-buffered saline (PBS) to remove excess unbound cells. Fixations of adherent cells were done with 200 μl of methanol for 20 min. Then the tubes were air-dried. The fixed air-dried bacterial biofilms were stained for 20 min with 200 μl of 1% crystal violet dissolved in distilled water. Excess stain was removed by washing the tubes with distilled water. Withdrawal of the cell-bound crystal violet was achieved by adding 200 μl of 30% glacial acetic acid prepared in distilled water, and the absorbance was taken at 595 nm using cell-free medium as a control.

### Statistical analysis.

All experiments were performed in parallel sets of triplicates. The values were calculated and are represented as the mean ± standard deviation (SD) (41). Statistical significance was calculated by analyzing paired *t* tests.

### Purification and characterization.

Purification of extracellular amylase was done by ammonium sulfate precipitation in cell-free broth (10). Precipitated enzyme was separated by centrifugation (5,000 rpm for 20 min) at 4°C and dissolved in 70 ml of 100 mM sodium phosphate buffer of pH 7.6. The semipurified enzyme was then dialyzed against the same buffer overnight at 4°C under stirring conditions (14). After centrifugation (15,000 rpm for 10 min) at 4°C, phenyl methane sulfonyl fluoride (PMSF) prepared in dimethyl sulfoxide (DMSO) (17 μg/ml) was added to the supernatant to concentrate up to 30 ml in a lyophilizer and stored at −20°C (14). To purify near homogeneity, the solution of bacillary enzyme was loaded on the DEAE cellulose column, which was equilibrated with 100 mM sodium phosphate buffer (pH 7.6), and for elution, the same buffer with an increasing gradient of sodium chloride (NaCl) concentrations (0.1 to 1.0 M) was used (42). The fractions with amylase (15 ml) were then pooled and lyophilized at 4°C. In each step, the protein content was determined using bovine serum albumin (BSA) as a standard (43).

The molecular weight of the purified amylase was determined by SDS-PAGE. A 12% gel was prepared following the method of Laemmli (17) and stained with Coomassie blue R250 prepared in methanol:acetic acid:water (4:1:5, vol/vol). The destaining solution used in this experiment has the same composition methanol:acetic acid:water (4:1:5, vol/vol) except Coomassie blue (27).

The effect of pH on the stability of amylase was evaluated by incubating 1 ml of the enzyme with 1 ml of starch solution (1%), for 24 h, prepared in the specific buffer to achieve different pHs (sodium acetate/phosphate/Tris-HCl) (5). However, the outcome of enzyme activity was measured by reacting enzyme (1 ml) and substrate solution (1% starch) prepared in the buffer (1 ml). To determine the effect of temperature on enzyme stability, the extracellular amylase was incubated up to 1 h at different temperatures and transferred immediately into ice before measuring the enzyme activity under standard assay conditions. The effect of metal ions on amylase activity was evaluated by adding corresponding salts into the reaction mixture to obtain a final concentration of 1 mM, followed by assay.

To determine the halotolerance, the enzyme solution was incubated with various concentrations of NaCl ranging from 1% to 21% for 30 min, before the enzyme assay was performed. However, to assess the type of amylase, a 2% starch solution was incubated with enzyme solution at room temperature for 15 min, and 2 ml of freshly prepared Lugol’s iodine solution was added to check the color change against standard α-amylase (Sigma). For end product analysis of the enzyme-substrate reaction, the paper chromatographic method was followed with Whatman no. 1 filter paper. The end products were visualized by spraying with aniline phthalate and baking at 105°C for 10 min in an oven (23).
